# Diversity, Genomics and Symbiotic Characteristics of Sinorhizobia That Nodulate *Desmanthus* spp. in Northwest Argentina

**DOI:** 10.3390/biology12070958

**Published:** 2023-07-04

**Authors:** Nicolás Emilio Zuber, Laura Viviana Fornasero, Sofía Agostina Erdozain Bagolín, Mauricio Javier Lozano, Juan Sanjuán, María Florencia Del Papa, Antonio Lagares

**Affiliations:** 1 IBBM—Instituto de Biotecnología y Biología Molecular, CONICET, CCT-La Plata, Departamento de Ciencias Biológicas, Facultad de Ciencias Exactas, Universidad Nacional de La Plata, Calles 47 y 115, La Plata 1900, Argentina; 2 Facultad de Ciencias Agrarias, Universidad Nacional del Litoral, Esperanza 3080, Argentina; 3 Departamento de Microbiología del Suelo y la Planta, Estación Experimental del Zaidín, Consejo Superior de Investigaciones Científicas (CSIC), E-18008 Granada, Spain

**Keywords:** *Desmanthus virgatus*, *Desmanthus paspalaceus*, *Sinorhizobium*, *Mesorhizobium*

## Abstract

**Simple Summary:**

“Rhizobia” are alpha- and beta-proteobacteria with the ability to associate in symbiosis with legumes, establishing complex root structures (nodules) where the process of biological N_2_-fixation takes place. The knowledge of these bacteria which efficiently associate with particular legumes is a key aspect in understanding the origin, diversification, practical use, and improvement of the symbiotic systems. In this work, we present the phenotypic and genomic characteristics of a collection of bacterial isolates of the genera *Sinorhizobium*, present in soils of northwest Argentina and able to associate with plants of the *Desmanthus virgatus* complex. Results showed an important genetic diversity among the local isolates (including different species), heterogeneous profiles of tolerance to abiotic stresses, and an outstanding capacity to support plant growth in the absence of fixed nitrogen. Upon the genomic and phylogenetic analyses of selected isolates, evidence of the horizontal gene transfer of nodulation (*nod*) genes was obtained, accounting for current genomic constitution of the local symbionts of *Desmanthus*.

**Abstract:**

*Desmanthus* spp. are legumes with the ability to associate with diverse α-proteobacteria—a microsymbiont—in order to establish nitrogen-fixing root nodules. A previous investigation from our laboratory revealed that the main bacteria associated with *Desmanthus paspalaceus* in symbiosis in central Argentina (Province of Santa Fe) were quite diverse and belonged to the genera *Rhizobium* and *Mesorhizobium*. To achieve a more extensive view of the local microsymbionts associated with *Desmanthus* spp., we sampled three different sites in Jujuy and Salta, in northwest Argentina. Matrix-assisted Laser-Desorption-Ionization Time-of-Flight mass spectrometry (MALDI-TOF) typing, 16S-rDNA analysis, and genome sequencing demonstrated that the dominant root-nodule microsymbionts belonged to the genus *Sinorhizobium*, with some sequenced genomes related to *Sinorhizobium mexicanum*, *Sinorhizobium chiapanecum*, and *Sinorhizobium psoraleae*. An analysis of *nodA* and *nodC* markers indicated that, in some of the isolates, horizontal gene transfer appeared to be responsible for the lack of congruence between the phylogenies of the chromosome and of the symbiotic region. These results revealed diverse evolutionary strategies for reaching the current *Desmanthus*-microsymbiont diversity. What is remarkable beside their observed genetic diversity is that the tolerance profiles of these isolates to abiotic stresses (temperature, salt concentration, pH) were quite coincident with the separation of the sinorhizobia according to place of origin, suggesting possible ecoedaphic adaptations. This observation, together with the higher aerial dry-weight matter that some isolates generated in *Desmanthus virgatus* cv. Marc when compared to the biomass generated by the commercial strain *Sinorhizobium terangae* CB3126, distinguish the collected sinorhizobia as constituting valuable germplasm for evaluation in local fields to select for more efficient symbiotic pairs.

## 1. Introduction

*Leguminosae* Juss. or *Fabaceae* Lindl. constitutes a vast and polymorphic plant family [1] comprising ca. 750 genera and ca. 20,000 species distributed in diverse ecosystems worldwide [2]. Furthermore, grain and forage legumes include many species that, in association with bacteria generically called “rhizobia” (i.e., the microsymbionts), have a central role in the incorporation of atmospheric nitrogen into natural and cultured soils [3,4]. In the example of forage legumes, their ecologic and commercial relevance comes from the quantity and quality of their contribution to ruminant diets [5], constituting a main incentive for undertaking intensive research to try to improve cultivar yields and symbiosis quality through the use of efficient rhizobia.

In recent years, several authors have reported the forage potential of the genus *Desmanthus* in livestock feeding [6,7,8,9]. On the basis of these investigations, several forage cultivars of different *Desmanthus* species have been released in Australia [10,11] and the USA. In particular, *Desmanthus virgatus* (*sensu lato*) is a taxonomic complex of plants in which four species native to north-central Argentina are currently recognized: *Desmanthus acuminatus* Benth., *Desmanthus paspalaceus* (Lindm.) Burkart, *D. virgatus* (L.) Willd., and *Desmanthus tatuhyensis* Hoehne [12,13]. The first three species have greater forage potential because of their leafiness, palatability, and reseeding capability [14,15,16]. The essential value of the local germplasm was indicated by several accessions of *D. virgatus*, *D. paspalaceus*, and *D. acuminatus* that have been evaluated in other countries, including the plant material from which the cultivar Marc, originally collected in Argentina, was developed [11]. *Desmanthus virgatus* and *D. paspalaceus* are perennial species with a spring-summer cycle that constitutes a promising forage alternative for use with livestock in marginally productive areas of Argentina. The positive characteristics of the species include: (a) high crude-protein contents (between 20 and 22% in leaves), (b) high yields that approach 35 tons dry matter/h/year [17], (c) resistance to drought, and (d) tolerance to the competition of herbaceous plants [15,18]. Though previous trials with these crops in Australia have reported symptoms of chlorosis, low vigor, and poor plant production; those problems were attributed to an inadequate nodulation [19], thus pointing to a need for more efficient N-fixing microsymbionts.

Several investigations have focused on the isolation and symbiotic characterization of rhizobial strains able to associate with *Desmanthus* spp. Date [20] employed rhizobia isolated from *Leucaena leucocephala* and *Neptunia plena* as field inoculants, with variable plant responses depending on the soil and the indigenous rhizobia that were present. The inoculation of *D. virgatus* with the *Rhizobium* strain CB3126 was reported to increase plant-leaf production relative to the uninoculated controls both in greenhouses [19] and in the field [21]. Though strain CB3126 had been recommended earlier as an inoculant for *D. virgatus* [20], more efficient microsymbionts were later found in selected root-nodule bacteria collected in soils from Santa Fe, Argentina [22]. In parallel, several authors collected several *Desmanthus* spp. in North, Central, and South America [6,7,9,23,24] that revealed the need to investigate the characteristics of the microsymbionts in the Americas. Thus, Beyhaut et al. [25] characterized root-nodule isolates from *Desmathus illinoensis* collected in the USA, which had been typified as *Rhizobium giardinii* (reclassified as *Pararhizobium girdinii* [26]) and which resulted non-infective on *D. virgatus*. Some years later, Fornasero et al. [22] reported the phenotypic, molecular, and symbiotic characterization of *D. paspalaceus* symbionts present in the soils of Santa Fe, Argentina. The isolates collected were remarkably diverse and were typified as *Rhizobium* and *Mesorhizobium,* contrasting with the inoculant strain CB3126 that was typified as *Sinorhizobium terangae*.

In order to gain a further insight into the nature and distribution of microsymbionts associated with *D. virgatus* and *D. paspalaceus*, we characterized root-nodule bacteria that originated from the soils of northwest Argentina. Isolates that proved to be mostly sinorhizobia, in contrast to all previous reports, provided symbiotically valuable germplasm, and contributed with novel evidence to the knowledge of the role of horizontal gene transfer in the evolution of microsymbionts of *Desmanthus* spp.

## 2. Materials and Methods

### 2.1. Isolation, Cultivation, and Preservation of Rhizobia from Northwest Argentina with the Ability to Nodulate Desmanthus spp.

Rhizobia with the ability to nodulate *Desmanthus* spp. were recovered from root nodules both collected in the field and from trapping plants in the laboratory, as previously described [22]. Root nodules were surface-sterilized as already reported Del Papa et al. [27] by immersion in 30 vol. H_2_O_2_ for 8 min, washed with distilled water, and crushed in 100 µL of Fåhraeus mineral solution [28]. Root-nodule bacteria were cultivated in yeast extract-mannitol (YEM) medium (g/liter: 0,5 yeast extract, 5 manitol, 0.5 K_2_HPO_4_, 0.2 MgSO_4_·7 H2O, 0.1 NaCl, pH 7) [29] and TY medium (g/liter: 5.0 tryptone, 3.0 yeast extract, 0.9 CaCl_2_·2 H_2_O, pH 7) [30] at 28 °C and 180 rpm. Bacterial clones were confirmed in their *Desmanthus* spp. nodulation phenotype and finally cryopreserved in 20% (*v*/*v*) glycerol stocks at −80 °C. Table 1 lists isolates recovered from *D. paspalaceus* and *D. virgatus* root nodules, with the corresponding place of origin (the provinces Salta and Jujuy in Argentina) and isolation strategy. The geolocations, together with the climatic and edaphic characteristics of the sampled soils in Jujuy and Salta, are presented in Table S1A,B

### 2.2. Evaluation of the Bacterial Tolerance to Abiotic Stresses That Are Frequently Present in Soils Populated with D. virgatus and D. paspalaceus

Bacteria isolated from the root nodules of *D. virgatus* and *D. paspalaceus* were evaluated in their ability to grow in agarized YEM media under the different stressing conditions frequently present in soils populated with *Desmanthus* spp. in northwest Argentina. An evaluation of the osmotic, pH, and temperature tolerances of the isolates was performed as described by [22]. Briefly, 10 μL of the bacterial suspensions containing ca. 10^4^ cells colony-forming units/mL were spotted onto YEM agar plates under each of the indicated conditions (Table S4), and the plates were then incubated at 28 °C until growth of the unstressed control. In all of these assays, the final bacterial growth was estimated on a scale of 0 to 5 (0, the absence of growth; 5, full development).

### 2.3. Typing and Analysis of Diversity in the Collection of Desmanthus-Nodulating Isolates

#### 2.3.1. Biotyping by Matrix-Assisted Laser Desorption-Ionization Time-of-Flight Mass Spectrometry (MALDI-TOF)

*Desmanthus*-nodulating bacteria were typified (at the genus level) by the MALDI-TOF analysis of whole-cell extracts by means of an Ultraflex III UV-MALDI-TOF/TOF mass spectrometer and Biotyper 3.1 software (Bruker Daltonics, Bremen, Germany) [31]. When necessary, an extended reference database was built up as previously reported by Ferreira et al. [32] and Sánchez-Juanes et al. [33] through the incorporation into the database of novel bacteria for which the genera had been previously deduced from a 16S-rDNA analysis. To obtain the MALDI-TOF spectra, bacterial isolates were cultivated in TY at 28 °C, and sample preparation carried out as previously reported by Toniutti et al. [34]. The procedure stated in brief: samples of 1 μL were overlaid with 1 µL of a saturated solution of α-cyano-4-hydroxycinnamic acid in 50% (*v*/*v*) acetonitrile and 2.5% (*v*/*v*) trifluoroacetic acid in water. The mass spectra were recorded in the range from 2 to 20 kDa with the Flex Control 3.3 software (Bruker Daltonics) in a positive linear mode at an accelerated voltage of 19 KV. For each spectrum, successive shots were collected to obtain data with maximal absolute peak intensities ranging from approximately 5 × 10^3^ to 1 × 10^4^ arbitrary units. An external calibration was performed with the Bruker bacterial test standard (Bruker Daltonics). Bacterial identification was performed by MALDI Biotyper Offline classification software through the use of the score values proposed by the manufacturer, with a score ≥2 indicating species identification, a score between 1.7 and 1.9 genus identification, and a score <1.7 no identification [32,35].

#### 2.3.2. Evaluation of Genomic Diversity by BOXA1R PCR-Fingerprint Analysis

Small-scale preparations of total DNAs were carried out according to Meade et al. [36] and used as polymerase chain-reaction (PCR) templates. Total DNA-amplification fingerprints were performed with the BOXA1R primer [37] under the cycling conditions previously described by Fornasero et al. (2014).

#### 2.3.3. Genomic Sequencing and Annotation of Selected Root-Nodule Bacteria

DNA samples of selected root-nodule isolates were prepared through the use of the AccuPrep Genomic DNA Extraction Kit (Bioneer) following the manufacturer’s protocol. Illumina sequencing was performed at SNPsaurus (Eugene, OR, USA) with an Illumina HiSeq 4000 instrument/device. The sequencing was carried out by means of Illumina paired-end libraries with 2 × 150-bp reads in order to reach a 60× read depth. The sequencing reads were assembled through the SPAdes [38]. The genome assemblies for isolates 6-70, 6-117, 7-81 and 7-89 have been deposited at the GenBank under the accession numbers, GCA_030124375.1, GCA_030124365.1, GCA_030124405.1, GCA_030124325.1, respectively. Genomes were annotated through the Prokaryotic Genome Annotation Pipeline (PGAP) [39] available at the National Center for Biotechnology Information).

### 2.4. Phylogenetic Analyses of Selected Root-Nodule Bacteria

The chromosomal and *nod* phylogenies of selected bacterial isolates present in soils of Jujuy and Salta and recovered from root nodules of *Desmanthus* spp. were investigated by means of the following analyses.

#### 2.4.1. Amplification of a Partial Sequence of the 16S rDNA

In order to explore the chromosomal taxonomic position of selected isolates, primers fD1 and rD1 of the PCR described by Weisburg et al. [40] were used with each isolate to amplify a 16S rDNA sequence spanning the positions homologous to nucleotides 78–1355 (1278 bp) of the 16S rDNA in *Sinorhizobium medicae* NBRC100384^T^ (GenBank AB681159). The partial 16S rDNA sequences for isolates 6-70, 6-117, 7-81 and 7-89 have been deposited at the GenBank under the accession numbers, OR143309, OR143308, OR143310, and OR143311, respectively. The Sanger sequences of the amplified fragments were compared with the EzBiocloud 16SrDNA database that contains curated sequences from type strains only (http://www.ezbiocloud.net/, accessed on 26 June 2023) [41]. Upon selection of relevant sequences, a multiple alignment was performed with ClustalW [42] and a phylogenetic tree inferred by means of MEGA X (Molecular Evolutionary Genetics Analysis version X) [43]. For the tree reconstruction, the statistical method of Maximum Likelihood was used with a bootstrap option of 1000 replications under the Tamura-Nei model.

#### 2.4.2. Analysis of Average Nucleotide Identity (ANIb) and Whole Genome Distance-Based Phylogenetic Tree

Draft genome sequences of selected isolates were compared through the use of the JSpeciesWS online server [44]. Whole-genome pairwise similarity metrics were calculated by means of an average nucleotide identity ANIb [45]. The whole genome distance-based phylogenetic tree was generated with the tools available at https://tygs.dsmz.de/ (TSGS, Type Strain Genome Server, accessed on 26 June 2023).

#### 2.4.3. Phylogeny of Nod Symbiotic Markers

*nodA* and *nodC* sequences were obtained from the draft genomes of selected isolates. Then, partial sequences of *nodA* covering the positions homologous to nucleotides 68–593 (526 bp) of the *nodA* in *Sinorhizobium meliloti* 1021 (GenBank ASM696) along with partial sequences of *nodC* covering nucleotides 317–1133 (816 bp) of the *nodC* in the same reference *S. meliloti* strain were used for the alignments. The partial *nodA* sequences for isolates 6-70, 6-117, 7-81 and 7-89 have been deposited at the GenBank under the accession numbers, QN222_17910, QN226_27220, QN224_28005, and QN219_32710, respectively. The partial *nodC* sequences for isolates 6-70, 6-117, 7-81 and 7-89 have been deposited at the GenBank under the accession numbers, QN222_17900, QN226_27210, QN224_27995, and QN219_32700, respectively. The sequences from the local isolates were then aligned with their homologs from different sinorhizobia with the program CLUSTAL W in the MEGA X package. Phylogenetic trees were inferred by Maximum Likelihood under the same conditions as indicated for the 16S analysis.

### 2.5. Nodulation Tests to Characterize the Symbiotic Performances of Selected Sinorhizobial Isolates from Jujuy and Salta

For the plant tests, seeds of *D. virgatus* cv Marc were boiled for 10 s in distilled water for coat disruption [46]. The seeds were then surface-sterilized with 96% (*v*/*v*) aqueous ethanol (1 min), and 3% (*w*/*v*) NaClO (5 min) [47]. The seeds were next rinsed with sterile distilled water and kept in water for 1 h for swelling. The seeds were finally germinated in 1% (*w*/*v*) water-agar plates and the resulting seedlings individually planted in plastic pots containing vermiculite and Jensen mineral solution [48]. To investigate the ability of selected rhizobial strains to support plant growth in the absence of fixed nitrogen, 15-day-old plants were inoculated with 10 mL of a sinorhizobial suspension containing ca. 10^7^ colony-forming units (c.f.u.)/mL in Jensen mineral solution. At the end of the assay, 6 to 12 plants/isolate were harvested. Uninoculated and N-fertilized plants served as controls. The N-fertilization was performed by adding to each pot 50 mL of 0.05% (*w*/*v*) KNO_3_ (70 ppm N) weekly for 60 days, resulting in ca. 30 mg N incorporated into each pot containing a single plant. The experiment was carried out in a plant-growth chamber at 28 °C with a 16 h light photoperiod. After harvesting, the number of nodules/plant, the biomass of nodules/plant, the dry weight of the aerial part/plant and the dry weight of the roots/plant, were determined. The data were subjected to an analysis of variance (ANOVA). When significant differences were detected, the results from the different treatments were compared by Fisher’s least significant difference (LSD) test.

### 2.6. Statistical Calculations

Statistical analyses were performed for all the components studied with RStudio software [49], where the homogeneity and normality of variances were analyzed before proceeding with the ANOVA. Next, the LSD test was used to define statistically similar groups (*p* < 0.05). The packages “car” [50], “agricolae” [51] and “ggplot2” version 3.3.2 [52] were used to plot the figures.

For the principal components analysis (PCA) of the stress-tolerance phenotypes, we used the package “stats” [53] “dplyr” [54], “magrittr” [55], and “FactoMineR” [56] with reference to the numerical-tolerance ranking of each isolate for each stress (variables).

## 3. Results

### 3.1. Sinorhizobium spp., the Most Abundant Desmanthus-Nodulating Rhizobia Isolated from Soils of the Provinces of Salta and Jujuy, Argentina: Diversity and Phenotypic Characteristics

**Figure 1:**
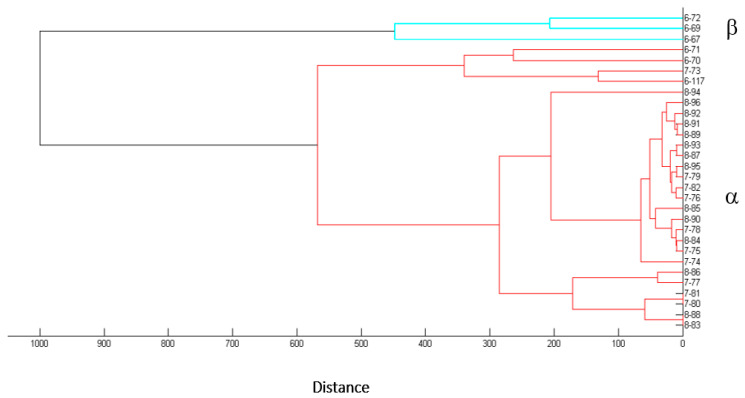
Dendrogram of mass-spectral profiles generated with the MALDI-TOF spectra collected for each of the indicated isolates. The distance dendrogram was generated through the BioTyper^TM^ software from Bruker. The distance levels are in relative units. Clusters α (red) and β (light blue) on the right side correspond to sinorhizobial and mesorizobial isolates, respectively, as detailed in the text.

**Figure 2:**
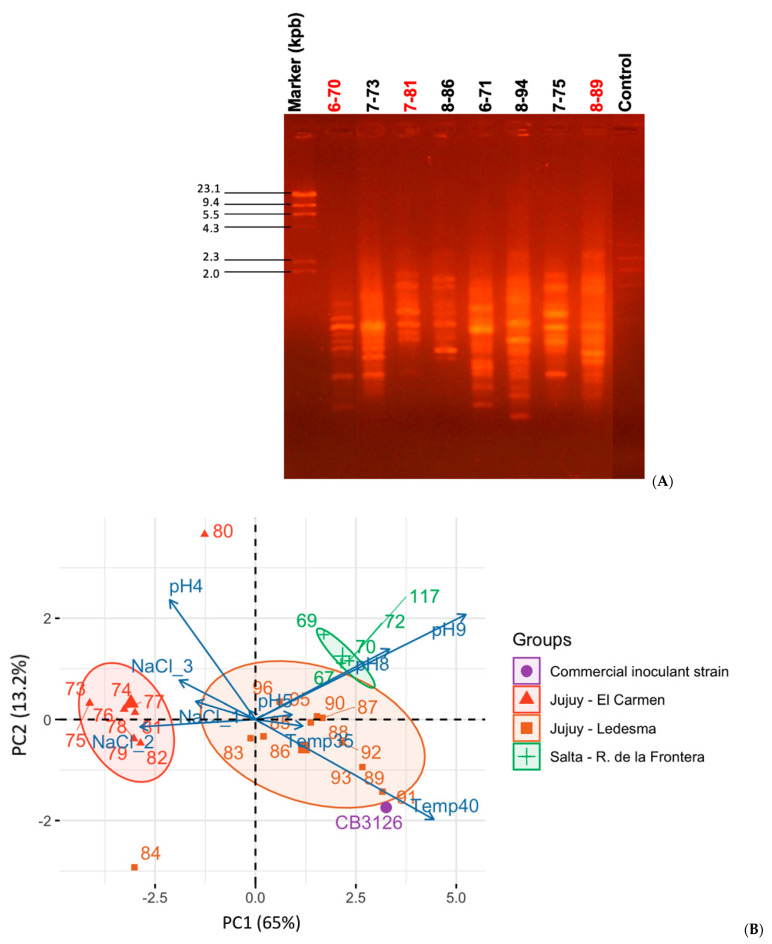
Genomic and phenotypic diversity of sinorhizobial isolates from northwest Argentina that nodulate *D. virgatus* and *D. paspalaceus*. (**A**) Genomic BOXA1R-PCR fingerprint patterns of bacterial isolates from Jujuy and Salta recovered from *D. virgatus* and *D. paspalaceus* root nodules (both field and laboratory isolates, cf. Table 1). Red numbers correspond to the isolates with sequenced genomes. (**B**) Principal component analysis (PCA)-based separation of isolates presented in Table 1 according to their differences in tolerance to abiotic stresses. The figure illustrates the distribution of the isolates in the first two components of variation, overlapping with a vector-correlation plot of the variables examined (i.e., stress tolerances) in the PC1-PC2 space of variation. The PCA analysis (R Studio software) was performed upon considering a numerical tolerance ranking for each isolate and stress (variables: temperature, salt concentration, pH). The distribution of bacteria in the PC1–PC2 space (representing more than 78% of the total variation) served to clearly separate isolates from Salta (in green; isolates from the sampling site 6, Table S1A) and Jujuy (in red; isolates sampling site 7 and in orange; sampling site 8, Table S1A) on the basis of their differential phenotypes. To improve clarity, in this plot designation of isolates does not include the reference to the site of origin (e.g., isolate 117 instead 6-117).

In order to isolate *Desmanthus*-nodulating rhizobia from soils of the provinces of Salta and Jujuy, Argentina, bacteria were recovered from root nodules collected from *Desmanthus* spp. in the field and also from nodules generated in the laboratory by planting trapping plants in soil samples following previously established protocols (Section 2, Fornasero et al. [22]). By using those procedures, 30 rhizobial isolates could be recovered: 3 from nodules collected in the field and 27 from nodules generated under laboratory conditions (Table 1, column 4). With the aim of grouping isolates at the genus and species level, we obtained MALDI-TOF spectra of bacterial-cell extracts (Section 2) that were then analyzed with the Biotyper (Brucker) software, as reported elsewhere [57]. Those mass spectra served to construct the distance-based tree in Figure 1. Two main groups of isolates were generated, namely α and β, which included 27 and 3 isolates, respectively. No isolates from the α and β-groups exhibited significant similarity to any reference strain from the Biotyper database. By using a specific PCR with the primer pair 16S4 and rD1 previously used in our laboratory [22], isolates from the β-group could be identified as related to *Mesorhizobium* spp. (not shown). Then, in order to explore the taxonomic position of isolates from the α-group, 1278 bp sequences of the 16S rDNAs from isolates 6-70, 6-117, 7-81, and 8-89 were obtained (*cf*. Section 2) and compared with the EZBioCloud database [41]. The results indicated that the four selected isolates corresponded to *Sinorhizobium* spp. (Table S2). An updating of the Biotyper database with this information then served to identify all the isolates from the α-group as *Sinorhizobum* spp. (Table 1, column 5, Table S3). The sinorhizobia thus proved to be the most abundant microsymbionts that nodulate *Desmanthus* in the soils sampled from the provinces of Jujuy and Salta. A BOXA1R [37] DNA fingerprinting performed on a subset of nine sinorhizobial isolates (Figure 2, Panel A) and aimed at exploring their genomic heterogeneity revealed the genetic diversity existing among the isolates collected (also among isolates from the same soil such as with the isolates 7-73 and 7-81, which differed in their BOXA1R profiles) (Figure 2A). A similar diversity was also reflected in the different growth responses of the sinorhizobia upon experiencing various challenging environmental conditions (such as high salt concentrations, pH stresses, and high temperature).

We performed a PCA using the growth scores that corresponded to each of the conditions studied (Table S4), with the overall results being illustrated in Figure 2, Panel B. The first two principal components (PCs) included 78% of the phenotypic variation, with PC1 and PC2 representing 65% and 13% of the observed variation, respectively. Beyond the existing genetic diversity reflected by the different BOXA1R-fingerprint patterns (Figure 2, Panel A), the remarkable feature is that, with only two exceptions (Isolate 7-80 and 8-84) all the isolates were grouped in the PCA according to their geographical origin (i.e., isolates from Salta within the green ellipse, and isolates from Jujuy within the red and brown ellipses). Nevertheless, the two outrider isolates were both from Jujuy as well. Whereas the isolates from Jujuy included several sinorhizobia with the ability to grow at either moderately high salt concentration (e.g., isolates 7-73 to 7-79 from sampling site 7) or high temperature (e.g., isolates 8-89, 8-91 from sampling site 8), the isolates from Salta clustered closer to each other, evidencing an ability to grow in moderately alkaline media (isolates 6-67–6-117).

The investigations presented in the next sections were focused on analyzing the taxonomic, genomic, and symbiotic characteristics of the sinorhizobial isolates 6-70, 6-117, 7-81, and 8-89 of the α group (Figure 1).

### 3.2. Taxonomic Position of Selected Desmanthus-Nodulating Sinorhizobial Isolates

**Figure 3:**
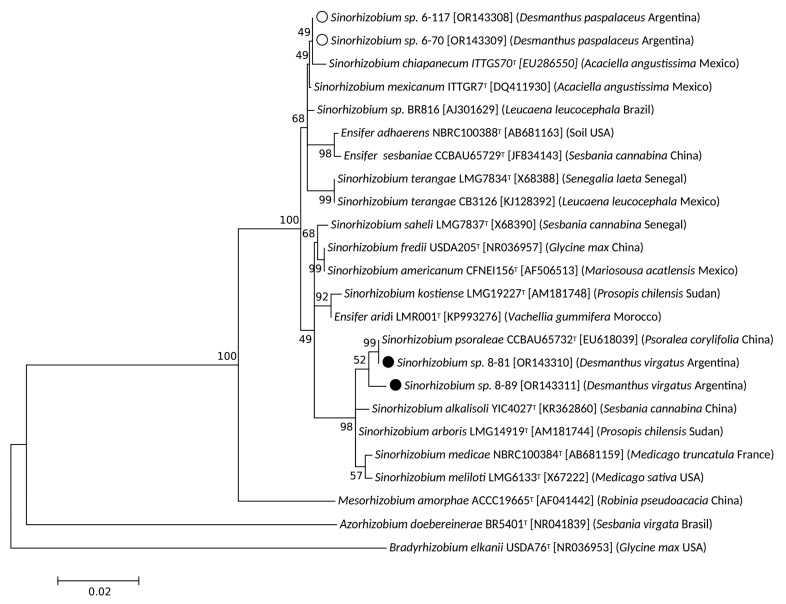
Phylogenetic (maximum-likelihood) tree illustrating the relationship between sinorhizobial 16S rDNA sequences, including the isolates recovered from *D. paspalaceus* and *D. virgatus* plants collected in Jujuy and Salta, Argentina. The phylogeny was reconstructed by using partial sequences of the 16S rDNA genes (*cf*. Section 2). Alignments were performed with sequence stretches that covered the positions homologous to nucleotides 78–1355 (1278 bp) of the 16S rDNA in *Sinorhizobium medicae* NBRC100384^T^ (GenBank AB681159). The DNA sequences used were obtained from the GenBank under the accession numbers indicated after the name of each sinorhizobium. The 16*S*-rDNA sequences were analyzed by the maximum-likelihood method through the use of the MEGA X software package. The bootstrap consensus tree inferred from 1000 replicates under the Tamura-Nei model was constructed to represent the evolutionary history of the genes analyzed. The percentage of replicate trees in which the associated taxa clustered together in the bootstrap test is designated next to the branches. Open and closed circles refer to isolates from Salta and Jujuy, respectively.

On the basis of the results of the previous section, two isolates from Salta, 6-70 and 6-117, and two isolates from Jujuy, 7-81 and 8-89, were selected for further analysis. These isolates were mapped at separate locations on the PC1-PC2 space in Figure 2B, thus representing sinorhizobia with different growth phenotypes. The 1278-bp sequence of the 16S rDNA from these isolates (*cf*. previous section) together with orthologous sequences from related sinorhizobia were used to construct the maximum-likelihood phylogenetic tree illustrated in Figure 3. Isolates 7-81 and 8-89 (labelled with closed circles) were both grouped particularly close to *Sinorhizobium psoraleae* CCBAU 65732 and in the same clade as *S. meliloti* LGM6133, *S. medicae* NBRC 100384, *Sinorhizobium arboris* LGM 14919, and *Sinorhizobium alkalisoli* YIC 4027. Isolates 6-70 and 6-117 (labelled with open circles) were grouped within another clade close to the positions of both *Sinorhizobium chiapanecum* ITTGS-70 and *Sinorhizobium mexicanum* ITTG-R7. All these sinorhizobia were also grouped to form a broader clade with: *Sinorhizobium* sp. BR816, with a clade that contained *S. terangae* CB3126, and with a clade that included *Ensifer adherens* NBRC100388 and *Ensifer sesbaniae* CCBAU 65729. In order to gain a more clearly defined taxonomic position of our local isolates, we obtained their complete genomic sequences. Then, to compare these isolates with their closer sinorhizobia the ANIb values were calculated through the inspection of sequences available in the Genbank (*cf*. Section 2). The results presented in Table 2 indicate that isolates 6-70 and 6-117 from Salta have between them an ANIb value of 99.95%, suggesting that they might belong to the same species. Lower ANIb values were obtained when isolates 6-70 and 6-117 were compared with the strains *S. mexicanum* ITTG-R7 (92.90) and *S. terangae* SEMIA6460 (88.98%) (no genome sequence is yet available for *S. chiapanecum*). The isolates from Jujuy 7-81 and 8-89 exhibited between them an ANIb value of 94.94%, suggesting that they might belong to different species. Furthermore, isolate 7-81 from Jujuy was associated with a higher ANIb value (96.26%) upon comparison with *S. psoraleae* CCBAU65732 than the one obtained upon comparison with isolate 8-89. We must finally remark that, though the sinorhizobial isolates from Salta 6-70 and 6-117 were very similar (ANIb 99.95 %), they were nevertheless taxonomically distant from the isolates from Jujuy 7-81 and 8-89, with which they have ANIb values lower than 87%. A whole genome distance-based phylogenetic analysis was performed (Figure S1) and results indicated that the four sequenced isolates most likely belong to three different species: a same species for isolates 6.70 and 6-117, and two different species in case of isolates 7-81 and 7-89. According to this result and to the ANIb values (Table 2), isolate 7-81 might belong to the *S.* *psoraleae* species.

### 3.3. Symbiotic Genes in Desmanthus-Nodulating Sinorhizobia and Nod-Gene Phylogenies

**Figure 4:**
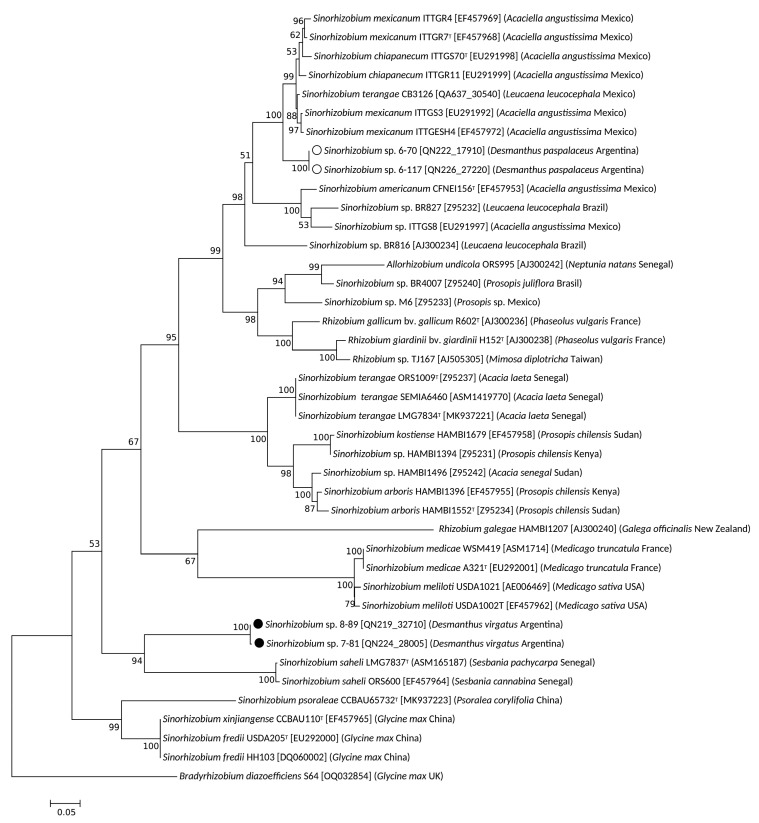
Phylogenetic (maximum-likelihood) tree illustrating the relationship between sinorhizobial *nodA* sequences, including the isolates recovered from *D. paspalaceus* and *D. virgatus* plants collected in Jujuy and Salta, Argentina. The phylogeny was reconstructed from partial sequences of *nodA* genes from different sinorhizobia (*cf*. Section 2). Alignments were performed with sequence stretches that covered the positions homologous to nucleotides 68–593 (526 bp) of the *nodA* in *Sinorhizobium meliloti* 1021 (GenBank ASM696). The DNA sequences used were obtained from GenBank under the accession numbers indicated after the name of each sinorhizobium. The *nodA* sequences were analyzed by the maximum-likelihood method through the use of the MEGA X software package as described in legend to Figure 3. Open and closed circles refer to isolates from Salta and Jujuy, respectively.

**Figure 5:**
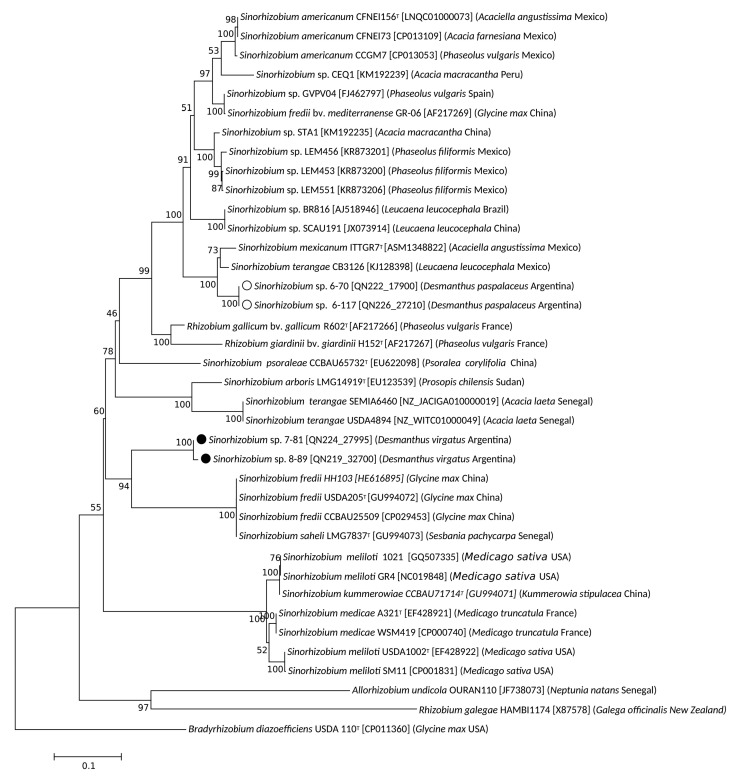
Phylogenetic (maximum-likelihood) tree illustrating the relationship between sinorhizobial *nodC* sequences, including the isolates recovered from *D. paspalaceus* and *D. virgatus* plants collected in Jujuy and Salta, Argentina. The phylogeny was reconstructed from partial sequences of *nodC* genes from different sinorhizobia (*cf*. Section 2). Alignments were performed with sequence stretches that covered the positions homologous to nucleotides 88–593 (816 bp) of the *nodC* in *Sinorhizobium meliloti* 1021 (GenBank ASM696). The DNA sequences used were obtained from GenBank under the accession numbers indicated after the name of each sinorhizobium. The *nodC* sequences were analyzed by the maximum-likelihood method through the use of the MEGA X software package as described in legend to Figure 3. Open and closed circles refer to isolates from Salta and Jujuy, respectively.

In order to explore evolutionary relationships of the nodulation functions in the isolates from Jujuy and Salta, we investigated their *nodA* and *nodC* phylogenies. A phylogenetic tree was constructed including isolates with *nodA* sequences that presented the highest sequence similarity to the *nodA*(s) from the four local sinorhizobia (Figure 4). Isolates 6-70 and 6-117 (labelled with open circles) clustered close to four strains of *S. mexicanum* and also close to two strains of *S. chiapanecum*, all of which nodulated *Acaciella agustissima* [58,59]. Thus, the *nodA* phylogeny of isolates 6-70 and 6-117 paralleled the 16S phylogeny and the genomic relationships inferred from the ANIb values presented in the previous section. Noteworthy, the *nodA* (Figure 4) and *nodC* (Figure 5) from the inoculant strain *S. terangae* CB3126 also clustered close to the homologs of the isolates from Salta, and not ―as it would be expected―at the position of the other *S. teranga* strains LMG7834 and ORS1009.

Contrasting with the previous patterns for the isolates from Salta, the *nodA* fragment from isolates 7-81 and 8-89 (labelled with closed circles) grouped with the homologous sequences of two strains of *Sinorhizobium saheli* that nodulated *Sesbania* and that were isolated in Senegal [60]. This group of *nodA* sequences did not evidence any strong relationship to other sequences in the *nodA* tree. The genomic similarity of isolates 7-89 and particularly 8-89 with *S. psoraleae* (ANIb values between 92% and 96%, respectively) did not correspond to a comparable phylogenetic relationship between their *nodA* symbiotic genes. This observation points to a likely horizontal acquisition of different symbiotic traits (different *nodA* alleles) from otherwise related chromosomal backgrounds (such as isolates, 7-81, 8-89 and *S. psoraleae*). For example, the *nodA* genes of isolates 7-81 and 8-89 are related to the *nodA* homolog from *S. saheli* LMG 7837 (Figure 4) despite the quite poor global genomic similarity of the latter strain with the isolates from Jujuy (ANIb 81.42 %, Table 2). It should be finally noted that the *nodA* phylogeny described here for the local isolates was mostly congruent with that of the *nodC* genes (Figure 5). The following difference, however, was observed: whereas the *nodA* alleles of isolates 7-81 or 8-89 and *S. saheli* did not exhibit a close relationship with their *nodA* homologs from *S. fredii*, the *nodC* from all these sinorhizobia grouped together with the high bootstrap value of 94% (Figure 5). Such observation suggests a possible ancestral recombination event between the *nodA* and *nodC* allels in *S. fredii*.

### 3.4. Symbiosis between the Sinorhizobial Isolates 6-70, 6-117, 7-81, or 8-89 and D. virgatus under Laboratory Conditions

**Figure 6:**
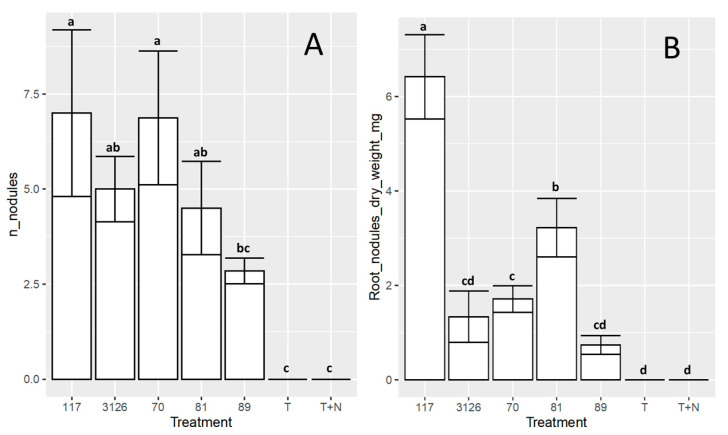

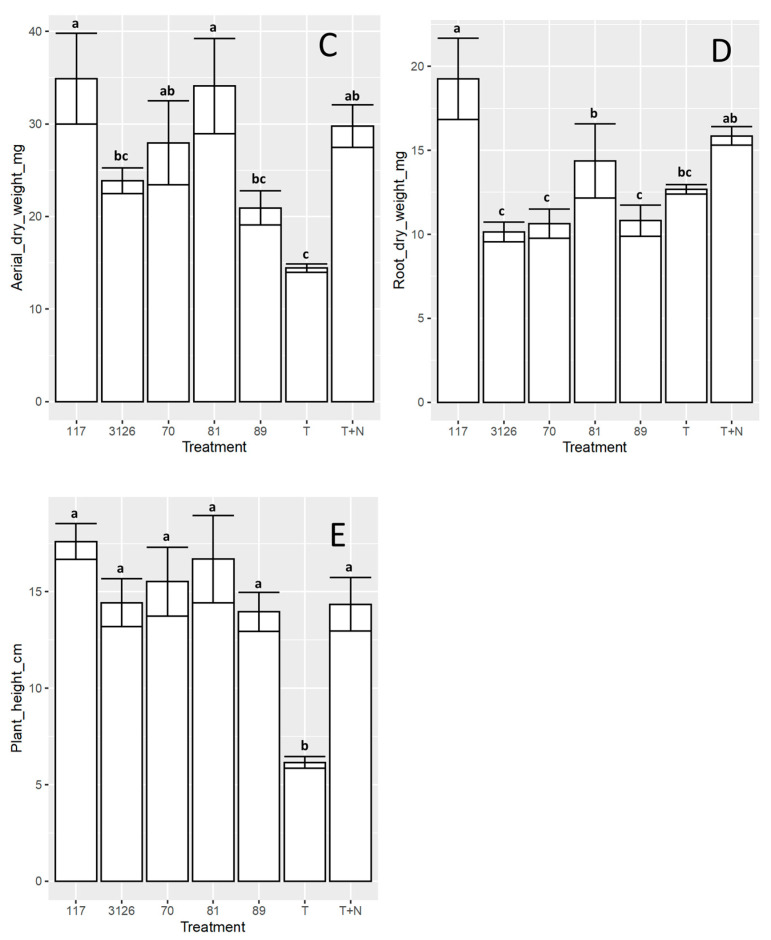
Effect of inoculation treatments on different plant-growth phenotypes when *D. virgatus* cv. Marc plants were grown in pots with vermiculite and mineral solution in a plant chamber. (**A**) Number of root nodules/plant. (**B**) Nodule biomass in mg/plant. (**C**) Aerial dry weight in mg/plant. (**D**) Root dry weight in mg/plant. (**E**) Plant height in cm. In each of the panels, the respective parameter was plotted on the ordinate for each of the experimental groups indicated on the abscissa. Plants of *D. virgatus* cv. Marc grown in plastic pots with N-free mineral solution and vermiculite were inoculated with the indicated rhizobia (Section 2). Uninoculated plants without N addition (negative control), and N-fertilized plants (positive control) were included in the experiment. The plants were harvested for analysis 60 days after inoculation. The values reported in the figure are the means of at least six individual plants. The bars represent the standard error (SE). Treatments with same letters are not significantly different according to Fisher’s LSD test (*p* = 0.05). To improve clarity designation of isolates does not include the reference to the site of origin (e.g., isolate 117 instead 6-117).

The ability of the isolates studied here to support the growth of *D. virgatus* cv. Marc in vermiculite with mineral solution and without an added N source was evaluated under laboratory conditions (*cf*. Section 2; Figure 6). Control plants without inoculation, uninoculated plants fertilized with nitrogen, and plants inoculated with the commercial strain *S. terangae* CB3126 were also included in the assay. The number and total weight of root nodules, the plant separate root and shoot dry weights, and the plant height were determined at 60 days post-inoculation. The results indicated that all the isolates, on average, generated between 4.5–7.0 nodules/plant root, except isolate 8-89 from Jujuy, which induced a lower number of root nodules (i.e., 2.8 nodules/plant root) than the value observed for the other sinorhizobia (Figure 6, Panel A). In addition to the number of nodules/plant, isolates 6-117 and 7-81 generated both a significantly higher nodule biomass than that observed in the other treatments, including strain CB3126 (Figure 6, Panel B). The results demonstrated that, although plant height was comparable in all the inoculated plants to that of the fertilized control (Figure 6, Panel E), isolate 6-117 and, to a lesser extent, isolate 7-81 likewise induced an aerial biomass that was comparable to that of the N-fertilized control, but higher than the value obtained for the commercial inoculant CB3126 (Figure 6C; ANOVA, *p* = 0.05). Consistent with this observation, the isolates 6-117 and 7-81, in that order, promoted the highest root biomass (Figure 6, Panel D) and also manifested the highest nodule biomass/plant (Figure 6, Panel B).

## 4. Discussion

Legumes belonging to the genus *Desmanthus* include several species for which different root-associated rhizobia have been described and with which those legumes establish either nitrogen-fixing nodules or ineffective symbioses. Beyhaut et al. [24,25] have reported that *D. illinoensis* in the USA could be efficiently nodulated by *Rhizobium giardini*, a species of rhizobia that was previously associated with the infection of *Phaseolus vulgaris*, *Leucaena leucocephala*, and *Macroptilium atropurpureum* [61]. More recent results have reported that mesorhizobia present in soils from the province of Santa Fe, Argentina efficiently nodulated *D. paspalaceus* and *D. virgatus* [22]. Thus, the current pattern indicates that *Desmanthus* spp. can be infected by several α-rhizobia with quite different genomic structures, where the host range to express an efficient nitrogen fixation may be restricted to the combination of a particular plant species and microsimbionts (e.g., the strains of *P. giardinii* isolated from *D. illinoensis* in the USA that were reported to be unable to fix nitrogen in *Desmanthus virgatus* [25]). Such a degree of selectivity indicates that the evolutionary divergence of *Desmanthus* at the species level was accompanied—at least in some instances—by the selection of different genera of microsymbionts to establish fully compatible associations.

In the present work, aiming to investigate the diversity of the symbioses with *Desmanthus virgatus* and *D. paspalaceus*, we addressed the reconstruction of phylogenetic relationships for a collection of local root-nodule bacteria isolated from northwest Argentina. The use of MALDI-TOF and 16S rDNA sequencing enabled us to conclude that 90% of the isolates from the provinces of Salta and Jujuy were sinorhizobia along with only 3% mesorhizobia. The ability of sinorhizobia to associate in symbiosis with *Desmanthus* spp. had been previously demonstrated by the example of strain CB3126, a bacterium originally recovered from root nodules of *L. leucocephala* [20] and that could be typified as *S. terangae* [22]. The high representation of sinorhizobia detected in Jujuy and Salta contrasted with the type of microsymbionts found in Santa Fe, Central Argentina, where mesorhizobia and rhizobia accounted for 60% and 40%, respectively, of the isolates recovered from *D. paspalaceus* root nodules (no sinorhizobia were detected) [22]. We do not know if such differences between the kind and proportion of microsymbionts recovered in Santa Fe and in the northwest region of Argentina might be a consequence of differential ecoedaphic factors that favored the prevalence of distinct root-nodule bacteria in each region. With respect to the sites in the northwest region studied here (two in Jujuy, one in Salta), the occurrence of an environmental selection of particular sinorhizobia could also be feasible, as revealed by the different tolerances to abiotic stresses which were evident among the isolates from the three different sampling sites (PCA analysis, Figure 2, Panel B). In regard to such a possibility, complementary studies will be necessary to evaluate whether the edaphic conditions at each site sampled do or do not correlate with the isolates’ phenotypes. The higher tolerance to pH 9 displayed by the sinorhizobia from Salta agrees, for example, with the slightly alkaline soils at that site (Table S1B). Though the profiles of stress tolerance were good predictors of the isolates’ site of origin; the simple tolerance to high salt, high temperature, and extreme pH conditions might only be the visible manifestation of more complex environment related adaptatons.

The genomic whole genome analysis (ANIb values in Table 2, phylogeny in Figure S1) of four selected sinorhizobia indicated that those isolates most likely belonged to three different sinorhizobial species. None of the isolates sequenced were identified as *S. terangae*, the species of strain CB3126. Whereas the two sinorhizobia from Salta (6-70 and 6-117) proved to be genetically highly similar and closely related to *S. chiapanecum* and *S. mexicanum* (isolated from *Acaciella agustissima* in Mexico) [58,59], the two isolates from Jujuy (7-81 and 8-89) had genomes related to *S. psoraleae* (isolated from *Psoralea campyfolia*); but the *nodA* and *nodC* of those isolates were related to a homolog in *S. saheli* (isolated from *Sesbania pachycarpa*) [60]. While the genomic and the *nod* phylogenies were congruent for the two isolates from Salta, a similar relationship was not observed for the isolates from Jujuy. The horizontal gene transfer of symbiotic and nonsymbiotic genes among rhizobia has been well documented in the literature [62,63,64]. The results presented here are consistent with the existence of a common genomic ancestor for the two isolates from Jujuy and for *S. psoraleae*, which should then have also acquired the corresponding *nod* information and the ability to nodulate *Desmanthus* and/or *Sesbania* and/or *Psoralea*. Neither the ability of the local isolates 7-81 and 8-89 to nodulate *Sesbania* or *Psoralea* nor that of isolates 6-70 and 6-117 to nodulate *Acacyella* has yet been evaluated.

The symbiotic characterization of the four selected isolates under laboratory conditions indicated that isolate 6-117 (from Salta) and 7-81 (from Jujuy) induced aerial biomasses that paralleled that of the N-fertilized control and gave rise to individuals that were taller than those observed with plants inoculated with the commercial inoculant strain CB3126 (Figure 6). The root-nodule bacteria collected in general, and isolates 6-117 and 7-81 in particular, constitute a valuable source of germplasm to be assessed for the inoculation of plants from the *D. virgatus* complex. From a practical viewpoint, in order to find symbiotic pairs that could express particular ecoedaphic adaptations, further studies should be conducted in the field to investigate different combinations of plant-sinorhizobia genotypes for each of the environments studied.

The results of the present work, together with those previously reported by Fornasero et al. [22], depict an intricate pattern for the symbioses between plant species of the *D. virgatus* complex and their root-nodulating bacteria. The microsymbionts present in Argentina include rhizobia, mesorhizobia, and sinorhizobia—members of three different bacterial genera. This raises several questions concerning the basic biochemical requirements that enable an efficient symbiosis to occur in *Desmanthus*: namely, whether, based on the different *nod* phylogenies observed, all types of rhizobia recognize the same plant signals as *nod* inducers; how diverse the chemistry of the resulting Nod factors is among the different micrsymbionts; whether *D. virgatus* and *D. paspalaceus* have particular preferences for a given microsymbiont (genera and/or species) over the others; and whether any relationship exists between the geographic distributions of the host plants (i.e., the particular *Desmanthus* spp.) and any particular rizo-/meso- and/or sinobacterial symbiont.

The results of this work provide a consolidated view on the biodiversity and symbiotic characteristics of the associations between the plants of the *Desmanthus virgatus* complex and their root-nodule bacteria, with a focus on the region of northwest Argentina. The new information and germplasm available will be useful to further investigate the biochemical bases and common features underlying such diverse symbiotic systems, as well as to screen for novel inoculant genotypes better adapted to specific environments.

## 5. Conclusions

In soils from northwest Argentina, sinorhizobia appear as the predominant nitrogen-fixing symbionts (90%) of the *D. virgatus* complex. The analysis of only four isolates―out of a total collection of 27 root-nodule sinorhizobia―revealed the presence of three different species, one of them being (or being close to) *S. psoraleae*. Among these sinorhizobia, at least two different classes of *nodA*/*nodC* genes were identified, one present in two isolates from Jujuy and the other in two isolates from Salta and in the inoculant strain *S. teranga* CB3126. The latter strain appeared as a symbiovar for *Desmanthus* when compared with other *S. terangae*. Taking all genetic information together, instances of horizontal gene transfer of *nod* information were evident, accounting for the current genomic structure of the *Desmanthus* symbionts. From a practical point of view, two of the isolates studied here―*Sinorhizobium* sp. 6-117 and 7-81―supported plant growth better than the inoculant strain CB3126, thus constituting optimal candidates for further studies in the field.

## Figures and Tables

**Table 1:** List of root nodule bacteria isolated from soils of northwest Argentina (provinces of Salta and Jujuy) through the use of *D. paspalaceus* and *D. virgatus* as trapping plants.

Isolate ID ^1^	Place of Origin	Natural Host	Origen of the Root Nodules	MALDI-TOF ^2^ Identification Group
6-67	Salta	*D. paspalaceus*	Trapping plant in the laboratory	β
6-69	Salta	*D. paspalaceus*	Trapping plant in the laboratory	β
6-70	Salta	*D. paspalaceus*	Trapping plant in the laboratory	α
6-71	Salta	*D. paspalaceus*	Trapping plant in the laboratory	α
6-72	Salta	*D. paspalaceus*	Trapping plant in the laboratory	β
6-117	Salta	*D. paspalaceus*	Trapping plant in the laboratory	α
7-73	Jujuy	*D. virgatus*	Trapping plant in the laboratory	α
7-74	Jujuy	*D. virgatus*	Trapping plant in the laboratory	α
7-75	Jujuy	*D. vitgatus*	Trapping plant in the laboratory	α
7-76	Jujuy	*D. virgatus*	Trapping plant in the laboratory	α
7-77	Jujuy	*D. virgatus*	Trapping plant in the laboratory	α
7-78	Jujuy	*D. vitgatus*	Trapping plant in the laboratory	α
7-79	Jujuy	*D. virgatus*	Trapping plant in the laboratory	α
7-80	Jujuy	*D. virgatus*	Plant collected in the field	α
7-81	Jujuy	*D. vitgatus*	Plant collected in the field	α
7-82	Jujuy	*D. virgatus*	Plant collected in the field	α
8-83	Jujuy	*D. virgatus*	Trapping plant in the laboratory	α
8-84	Jujuy	*D. vitgatus*	Trapping plant in the laboratory	α
8-85	Jujuy	*D. virgatus*	Trapping plant in the laboratory	α
8-86	Jujuy	*D. virgatus*	Trapping plant in the laboratory	α
8-87	Jujuy	*D. vitgatus*	Trapping plant in the laboratory	α
8-88	Jujuy	*D. virgatus*	Trapping plant in the laboratory	α
8-89	Jujuy	*D. virgatus*	Trapping plant in the laboratory	α
8-90	Jujuy	*D. vitgatus*	Trapping plant in the laboratory	α
8-91	Jujuy	*D. virgatus*	Trapping plant in the laboratory	α
8-92	Jujuy	*D. virgatus*	Trapping plant in the laboratory	α
8-93	Jujuy	*D. vitgatus*	Trapping plant in the laboratory	α
8-94	Jujuy	*D. virgatus*	Trapping plant in the laboratory	α
8-95	Jujuy	*D. virgatus*	Trapping plant in the laboratory	α
8-96	Jujuy	*D. vitgatus*	Trapping plant in the laboratory	α

^1^: Isolates’ IDs refer to: [sampled site]-[isolate number]. For geolocation of each sampled site see Table S1A. ^2^: Matrix-assisted laser desorption ionization time-of-flight mass spectrometry.

**Table 2:** Average nucleotide identity (ANIb) of four selected *D. virgatus*- and *D. paspalaceus*-nodulating sinorhizobia from northwest Argentina in comparison to their more related sinorhizobial type strains.

Average Nucleotide Identity (ANIb) (%)				
Sinorhizobial Strains ^1^	Sinorhizobia from Northwest Argentina Which Nodulate *Desmanthus* spp.			
	6-70 ^2^	6-117 ^2^	7-81 ^3^	8-89 ^3^
***Sinorhizobium alkalisoli* YIC4027** **^T^** **[GCA_008932245.1]**	79.64	79.66	79.92	79.89
***Sinorhizobium aridi* LMR001** **^T^** **[GCA_002078505.1]**	80.84	81.55	81.57	80.84
***Sinorhizobium mexicanum*** **ITTG R7^T^** **[GCA_013488225.1]**	**92.90** **^4^**	**92.90** **^4^**	85.97	85.95
***Sinorhizobium psoraleae*** **CCBAU 65732^T^** **[GCA_013283645.1]**	86.06	86.07	**96.29** **^4^**	**92.90** **^4^**
***Ensifer sesbaniae* CCBAU 65729** **^T^** **[GCA_013283665.1]**	78.77	78.77	78.83	78.77
***Sinorhizobium americanum* CFNEI 156** **^T^** **[GCA_001651855.1]**	80.48	80.51	80.32	80.66
***Sinorhizobium arboris* LMG 14919** **^T^** **[GCA_000427465.1]**	79.70	79.79	80.35	80.14
***Sinorhizobium saheli* LMG 7837** **^T^** **[GCA_001651875.1]**	81.16	81.18	81.42	81.40
***Sinorhizobium terangae* SEMIA 6460** **^T^** **[GCA_014197705.1]**	88.98	88.98	86.51	86.48
***Sinorhizobium terangae* CB3126** **[GCA_029714365.1]**	89.46	89.45	86.82	86.71
***Sinorhizobium* sp. 6-70** **^2^** **[GCA_030124375.1]**	-	99.99	86.66	86.64
***Sinorhizobium* sp. 6-117** **^2^** **[GCA_030124365.1]**	99.95	-	86.70	86.71
***Sinorhizobium* sp. 7-81** **^3^** **[GCA_030124405.1]**	86.47	86.48	-	95.24
***Sinorhizobium* sp. 8-89** **^3^** **[GCA_030124325.1]**	86.54	86.55	94.15	-

^1^: GenBank accession numbers for the genomic sequence of each strain are provided in brackets. ^2^: Isolate of soil from the province of Salta (see geolocation in Table S1A). ^3^: Isolate of soil from the province of Jujuy (see geolocation in Table S1A). ^4^: In bold are indicated the highest ANIb score against type strains for each of the four local isolates analyzed.

## Data Availability

Not applicable.

## Supplementary Materials

The following supporting information can be downloaded at: https://www.mdpi.com/article/10.3390/biology12070958/s1, Figure S1: Phylogenetic tree inferred with FastME from GBDP distances calculated from genome sequences; Table S1A: Geolocation of the sites sampled in the provinces of Salta and Jujuy and the host plant species at those sites; Table S1B: Climatic and edaphic characteristics of the sampled sites; Table S2: Best hits resulting from the EZBiocloud searches against partial 16S rDNA sequences of isolates 6-70, 6-117, 7-81 and 8-89; Table S3. MALDI-TOF typing of bacteria from root nodules of *D. virgatus* and *D. paspalaceus* which were nodulated in the field (Jujuy and Salta) or in the laboratory with soil samples; Table S4. Growth of rhizobia that nodulate *D. virgatus* and *D. paspalaceus* evaluated under different abiotic stressing conditions in agarized-YEM medium.
