# Supplementary material for: Diversity, Genomics and Symbiotic Characteristics of Sinorhizobia That Nodulate Desmanthus spp. in Northwest Argentina
Source: Biology (Basel). 2023 Jul 4;12(7):958. doi: 10.3390/biology12070958 (PMC10376216; doi:10.3390/biology12070958)
Supplement: Supplementary file 1 [file biology-12-00958-s001.zip › biology-2447258-supplementary.pdf]

Table S1A. Geolocation of the sites sampled in the provinces of Salta and Jujuy and the host plant species at those sites.

Sampled Site	ID [a]	Province	Department	GPS coordinates	Plant-host species
6	6995	Salta	Rosario de la Frontera	64° 56’ 40,8’W<br>25° 45’ 17,7’S	*D. paspalaceus*
7	7005	Jujuy	El carmen	65° 04’ 50,7’W<br>24° 32’ 15,15’S	*D. virgatus*
8	7059	Jujuy	Ledesma	64° 43’ 21,8’W<br>23° 45’ 40,1’S	*D. virgatus*

a: Corresponding collection number in the catalog of Dr. Pensiero, Facultad de Ciencias Agrarias, Universidad Nacional del Litoral, Argentina.

Tabla S1B. Climatic and edaphic characteristics of the sampled sites.

Sampled site [a]	Climatic data		Edaphic data						
					Chemical properties				
	Annual rainfalll (mm)	Mean Temperature (ºC)	Soil order[b]	Texture	OM[c] (%)	TON[d] (%)	AP[e] (ppm)	pH[f]	EC[g] (dS/m)
6	924.9	19.2	Entisols	sandy-gravelly	1.99	0.091	7	7.2	0.145
7	742.9	17.8	Molisols	Loamy-clay-loam	2.59	0.123	23	6.8	0.089
8	761.9	18.2	Molisols	Clay loam	2.11	0.100	17	6.9	0.105

a: Geolocation of the host plant species are detailed in Table S1.
b: Soil Taxonomy Classification System **c:** MO: Organic Matter **d:** TON: Total Organic Nitrogen
e: AP: Available phosphorus **f:** pH (soil:water ratio, 1:2) **g:** EC:Electrical conductivity.

Table S2. Best hits resulting from the EZBiocloud searches against partial 16S rDNA sequences of isolates 6-70, 6-117, 7-81 and 8-89.

Local isolate 16S rDNA query [1]	Best hit in EZBiocloud	Identity (%)
**6-70 (Salta)**	*Sinorhizobium mexicanum* ITTG R7	99.93
	Sinorhizobium chiapanecum ITTG S70	99.71
**6-117 (Salta)**	*Sinorhizobium mexicanum* ITTG R7	99.61
	Sinorhizobium chiapanecum ITTG S70	99.37
**7-81 (Jujuy)**	*Sinorhizobium psoraleae* CCBAU 65732	100.00
**8-89 (Jujuy)**	*Sinorhizobium arboris* LMG 14919	97.64

[1] Query sequence for each isolate corresponding to the sequence stretch that covered the positions homologous to nucleotides 78–1,355 (1,278 bp) of the 16S rDNA in *Sinorhizobium medicae* NBRC100384^{T} (GenBank AB681159).
The parentheses indicate the province of origen for each isolate (*cf*. the information of the site sampled in Table S1).

Table S3. MALDI-TOF typing of bacteria from root nodules of *D. virgatus* and *D. paspalaceus* which were nodulated in the field (Jujuy and Salta) or in the laboratory with soil samples.

Isolate name	Best candidate in data base	Biotyper Score [1]	Group [2]	Identification
**6-70**	6-70	2.84	α	*Sinorhizobium* spp.
**6-71**	6-70	1.9	α	*Sinorhizobium* spp.
**6-117**	6-117	2.84	α	*Sinorhizobium* spp.
**7-73**	6-117	2.01	α	*Sinorhizobium* spp.
**7-74**	8-89	2.423	α	*Sinorhizobium* spp.
**7-75**	7-81	2.088	α	*Sinorhizobium* spp.
**7-76**	8-89	2.488	α	*Sinorhizobium* spp.
**7-77**	7-81	2.138	α	*Sinorhizobium* spp.
**7-78**	8-89	2.155	α	*Sinorhizobium* spp.
**7-79**	8-89	2.548	α	*Sinorhizobium* spp.
**7-80**	7-81	2.41	α	*Sinorhizobium* spp.
**7-81**	7-81	2.84	α	*Sinorhizobium* spp.
**7-82**	8-89	2.46	α	*Sinorhizobium* spp.
**8-83**	7-81	2.55	α	*Sinorhizobium* spp.
**8-84**	8-89	2.13	α	*Sinorhizobium* spp.
**8-85**	8-89	2.10	α	*Sinorhizobium* spp.
**8-86**	8-89	1.70	α	*Sinorhizobium* spp.
**8-87**	8-89	2.52	α	*Sinorhizobium* spp.
**8-88**	7-81	2.15	α	*Sinorhizobium* spp.
**8-89**	8-89	2.85	α	*Sinorhizobium* spp.
**8-90**	8-89	2.08	α	*Sinorhizobium* spp.
**8-91**	8-89	2.58	α	*Sinorhizobium* spp.
**8-92**	8-89	2.45	α	*Sinorhizobium* spp.
**8-93**	8-89	2.58	α	*Sinorhizobium* spp.
**8-94**	6-70	1.85	α	*Sinorhizobium* spp.
**8-95**	8-89	2.56	α	*Sinorhizobium* spp.
**8-96**	7-81	1.73	α	*Sinorhizobium* spp.

1: Bacterial identification was performed with the MALDI Biotyper Software (Bruker) through the use of the score values proposed by the manufacturer, with a score ≥2 indicating species identification (green), a score between 1.7 and 1.9 genus identification (yellow), and a score < 1.7 no identification [30, 33].

2: *Cf*. See manuscript text, first section in Results and Fig. 1.

Table S4. Growth of rhizobia that nodulate *D. virgatus* and *D. paspalaceus* evaluated under different abiotic stressing conditions in agarized-YEM medium.

Isolate	Growth at the indicated temperature (°C) [1]			Growth at indicated pH [1,2]						Growth at NaCl (%w/v) concentration [1]				Province
	28	35	40	4	5	6	7	8	9	0.5%	1%	2%	3%	
**6-67**	5	5	2	2	2	5	5	5	5	5	4	0	0	Salta
**6-69**	5	4	1	2	4	5	5	5	5	5	4	0	0	Salta
**6-70**	5	5	2	2	4	5	5	5	5	5	4	0	0	Salta
**6-71**	5	5	2	2	4	5	5	5	5	5	4	0	0	Salta
**6-72**	5	5	2	2	4	5	5	5	5	5	4	0	0	Salta
**6-117**	5	5	2	2	4	5	5	5	5	5	4	0	0	Salta
**7-73**	5	4	0	3	3	5	5	3	1	5	5	4	2	Jujuy
**7-74**	5	4	0	3	3	5	5	3	1	5	5	2	1	Jujuy
**7-75**	5	4	0	3	3	5	5	3	1	5	5	4	2	Jujuy
**7-76**	5	4	0	2	3	5	5	3	1	5	5	2	1	Jujuy
**7-77**	5	4	0	3	3	5	5	3	1	5	5	2	1	Jujuy
**7-78**	5	4	0	2	3	5	5	3	1	5	5	2	1	Jujuy
**7-79**	5	4	0	2	3	5	5	3	1	5	5	2	1	Jujuy
**7-80**	5	5	0	5	5	5	5	5	4	5	4	3	2	Jujuy
**7-81**	5	4	0	3	4	5	5	3	1	5	4	2	1	Jujuy
**7-82**	5	4	0	2	3	5	5	3	1	5	4	2	1	Jujuy
**8-83**	5	5	1	1	4	5	5	4	3	5	4	2	0	Jujuy
**8-84**	5	5	1	1	4	5	5	1	0	5	4	2	0	Jujuy
**8-85**	5	5	1	1	4	5	5	4	3	5	4	1	0	Jujuy
**8-86**	5	5	1	1	4	5	5	4	3	5	4	1	0	Jujuy
**8-87**	5	5	2	1	4	5	5	5	4	5	4	1	0	Jujuy
**8-88**	5	5	2	1	4	5	5	5	4	5	4	1	0	Jujuy
**8-89**	5	5	5	1	4	5	5	5	4	5	4	1	0	Jujuy
**8-90**	5	5	2	1	4	5	5	5	4	5	4	1	0	Jujuy
**8-91**	5	5	5	1	4	5	5	5	4	5	4	1	0	Jujuy
**8-92**	5	5	3	1	4	5	5	5	4	5	4	1	0	Jujuy
**8-93**	5	5	4	1	4	5	5	5	4	5	4	1	0	Jujuy
**8-94**	5	4	2	1	4	5	5	5	4	5	3	2	0	Jujuy
**8-95**	5	4	2	1	4	5	5	5	4	5	4	1	0	Jujuy
**8-96**	5	5	0	0	1	5	5	5	4	5	4	1	0	Jujuy
**CB3126**	5	5	4	0	4	5	5	5	4	4	2	1	0	Commercial inoculant

1: Scores from 5 to 0 indicate the ability of rhizobia to grow under the condition investigated (5 = full development in 2 days, 0 = absence of growth).

2: YEM medium at pH 4 was softer, as expected, than the medium at pH 7 but stable enough to properly streak isolates on the surface

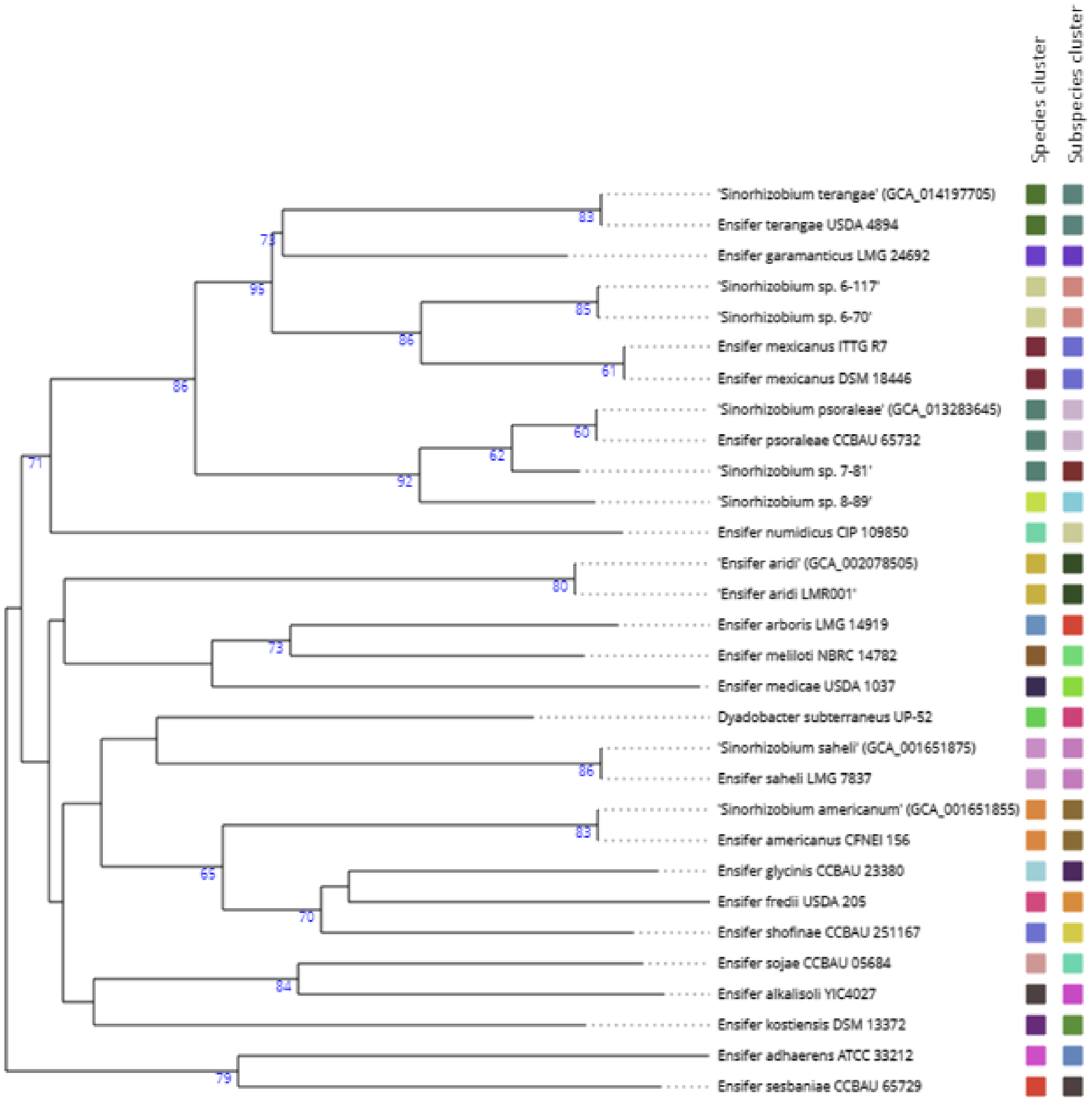

Figure S1. Phylogenetic tree inferred with FastME from GBDP distances calculated from genome sequences. The tree was generated with the tools available at https://tygs.dsmz.de/ (TSGS, Type Strain Genome Server). GBDP refers to genome Blast distances and FastME is an accurate and fast distance-based phylogeny inference program (http://www.atgc-montpellier.fr/fastme/). The numbers above branches are GBDP pseudo-bootstrap support values > 60 % from 100 replications. The tree was rooted at the midpoint.

Results indicate that the four sequenced isolates most likely belong to three different species: a same species for isolates 6.70 and 6-117, and two different species in case of isolates 7-81 and 7-89) (see color boxes at the first column on the right side of the tree). According to this result and to the ANIb values (Table 2), isolate 7-81 might thus belong to the *S. psoraleae* species.
